# Formation of pure Cu nanocrystals upon post-growth annealing of Cu–C material obtained from focused electron beam induced deposition: comparison of different methods

**DOI:** 10.3762/bjnano.6.156

**Published:** 2015-07-13

**Authors:** Aleksandra Szkudlarek, Alfredo Rodrigues Vaz, Yucheng Zhang, Andrzej Rudkowski, Czesław Kapusta, Rolf Erni, Stanislav Moshkalev, Ivo Utke

**Affiliations:** 1 Empa, Swiss Federal Laboratories for Materials Science and Technology, Laboratory for Mechanics of Materials and Nanostructures, Feuerwerkerstrasse 39, 3602 Thun, Switzerland; 2 AGH University of Science and Technology, Academic Centre for Materials and Nanotechnology, al. A. Mickiewicza 30, 30-059 Krakow, Poland; 3 Center for Semiconductor Components, State University of Campinas, 13083-870, Campinas, SP, Brazil; 4 Empa, Swiss Federal Laboratories for Materials Science and Technology, Electron Microscopy Center, Überlandstrasse 129, 8600 Dübendorf, Switzerland; 5 AGH University of Science and Technology, Faculty of Physics and Applied Computer Science, Department of Solid State Physics, al. A. Mickiewicza 30, 30-059 Krakow, Poland

**Keywords:** Cu(hfac)_2_, Cu nanocrystals, focused electron beam induced deposition (FEBID), post-growth annealing of Cu–C material

## Abstract

In this paper we study in detail the post-growth annealing of a copper-containing material deposited with focused electron beam induced deposition (FEBID). The organometallic precursor Cu(II)(hfac)_2_ was used for deposition and the results were compared to that of compared to earlier experiments with (hfac)Cu(I)(VTMS) and (hfac)Cu(I)(DMB). Transmission electron microscopy revealed the deposition of amorphous material from Cu(II)(hfac)_2_. In contrast, as-deposited material from (hfac)Cu(I)(VTMS) and (hfac)Cu(I)(DMB) was nano-composite with Cu nanocrystals dispersed in a carbonaceous matrix. After annealing at around 150–200 °C all deposits showed the formation of pure Cu nanocrystals at the outer surface of the initial deposit due to the migration of Cu atoms from the carbonaceous matrix containing the elements carbon, oxygen, and fluorine. Post-irradiation of deposits with 200 keV electrons in a transmission electron microscope favored the formation of Cu nanocrystals within the carbonaceous matrix of freestanding rods and suppressed the formation on their surface. Electrical four-point measurements on FEBID lines from Cu(hfac)_2_ showed five orders of magnitude improvement in conductivity when being annealed conventionally and by laser-induced heating in the scanning electron microscope chamber.

## Introduction

Focused electron beam induced deposition (FEBID) is a direct maskless nanolithography technique, based on the local dissociation of adsorbates upon the irradiation with electrons [1]. The molecules are delivered into the microscope chamber by a gas injection system (GIS) where they reversibly physisorb onto the substrate surface. Part of the energy from the primary electron beam or from the secondary electrons generated in the vicinity of the impinging primary beam is transferred to the adsorbates and breaks their chemical bonds. The non-volatile fragments stick to the substrate surface whereas the volatile fragments are removed from the chamber by the pumping system. By controlling the beam scanning three dimensional structures of a complex shape can be created in a single direct-write deposition step onto planar or non-planar surfaces [2].

Nanodevices with various functionalities have been deposited comprising gas sensors [3–4], magnetic sensors [5–6] strain sensors [7], thermal sensors [8], photodetectors [9], and mode stabilizers for vertical surface emitting lasers [10]. Other deposits were used as ferromagnetic wires [11–12], superconducting wires [13], plasmonic structures [14], or as electrode nanocontacts [15–16]. The feasibility of obtaining 3D nanostructures with a high aspect ratio makes FEBID suitable for fabrication of high resolution probes to scanning magnetic force microscopy (MFM) [17–19].

### Purification methods of FEBID structures

For FEBID direct-write nanostructures lateral resolution can be well-controlled by adjusting the beam and gas flow settings [20] as well as by optimizing scanning strategies [21]. However, the purity of FEBID materials obtained with organic precursors still remains an issue. Recently, post-growth purification methods using electron beam irradiation in combination with thermal annealing and co-injection of reactive gases/ions were developed. In the case of Pt–C deposits, the catalytic properties of Pt nanoparticles facilitate the process of molecular oxygen dissociation, thereby increasing the efficiency of removing the carbonaceous matrix [22]. Pure Pt material was obtained with a post deposition treatment using O_2_ gas and a) laser pulsing [23] or b) low-temperature substrate annealing (up to 50 °C) [24]. The presence of H_2_O during electron irradiation performed at rt allowed for a total elimination of carbon from Pt–C deposits without affecting the shape [25]. The combination of thermal heating to 300 °C, injection of H_2_ gas and simultaneous electron irradiation led to pure Co deposits [26]. Microstructural changes were observed upon simple 5 keV electron beam curing of FEBID structures. The Pt–C deposits exhibited an increased conductivity by three to four orders of magnitude [27–28]. For W–C deposits an improvement of one order of magnitude was found [29].

Conventional post-growth annealing of FEBID material in vacuum was summarized in a review by Botman et al. [30]: The thermal energy which is delivered to the sample can cause a desorption of carbonaceous fragments increasing the metal concentration from 15 atom % of Au (rt) to 24 atom % of Au (at 100 °C). Increasing the substrate temperature during FEBID also favors the desorption of non-metallic dissociation by-products as it was observed by Mulders et al. [31] for various precursors: TEOS (tetraethylorthosilicate), Co(CO)_3_NO, Co_2_(CO)_8_, and Me_2_Au(acac) with the best purity enhancement for W(CO)_6_ (from 37 atom % at 25 °C to 59 atom % of W at 280 °C). However, the temperature rise during the deposition may not be favorable as it also decreases the residence time of adsorbates, significantly lowering the growth rate. Furthermore, the high temperature may also cause the diffusion of deposit atoms into or from the substrate.

A compromise approach is based on pulsed heating with an IR laser as a heat source which can generate abrupt temperature peaks in the microseconds range only (sufficient for desorption), allowing the substrate surface to equilibrate quickly and to replenish with new adsorbates before the next electron-beam scan frame. FEBID together with synchronized pulsed IR laser heating helped to increase the metal concentration of Au, W, Pt in FEBID deposits, however, did not fully remove the carbon. For deposits obtained from Me_2_Au(acac) the initial atomic ratio of C to Au decreased from 4 to 0.5 with the laser assistance [32]. For W(CO)_6_ FEBID the atomic ratio of W to C was improved from 1:4 to 2:1 [33]. For MePtCpMe_3_ FEBID the Pt concentration increased from about 15 atom % to 35 atom % [34]. Such an improvement was not observed when using a conventional heating stage during the deposition process with MePtCpMe_3_. The deposit obtained at 350 °C did not exhibit a different Pt/C ratio than the deposit obtained at room temperature, where 15 atom % Pt were measured [31].

### Copper purification

The very high conductivity of copper makes the localized deposition of this metal very attractive for applications in nano-electronics. The organometallic hexafluoroacetylacetonate (hfac)-based Cu(I) and Cu(II) precursors are widely used in chemical vapor deposition (CVD) methods due to their stability and high vapor pressure. They allow to obtain pure metal CVD films with the same resistivity as in a bulk material at deposition temperatures below 300 °C [35–37].

Recently, it was shown for condensed monolayers of Cu(hfac)_2_ (also Pt(hfac)_2_ and Pd(hfac)_2_) that electron-beam irradiation results in about 80 atom % of carbon content [38]. The metal content could be then increased by two sequential purification steps: 1) deposit bombardment with atomic oxygen 2) deposit bombardment with atomic hydrogen. In the first step the carbonaceous material was fully removed from the material and in the next step the metal oxide was reduced to the metal. Although this method was successfully applied to obtain a deposit with high metal purity, the total exposure time was rather long: 40 h for oxygen and 2 h for hydrogen. The efficiency of atomic hydrogen only for purification of Cu–C material obtained by an ion-induced deposition process at room temperature was shown by Chiang et al. [36]. It led to 99 atom % pure Cu films. H_2_/Ar microplasma-assisted FEBID increased the Cu content from 12 atom % to 41 atom % but also caused extended halo deposits [39]. Ga^+^ ion beam deposition showed that heating the substrate surface has a crucial influence on the properties of the deposit structure, from small isolated nanocrystals of Cu (ca. 20 nm) at 25 °C towards continuous thin films of pure copper at 100 °C, using (hfac)CuVTMS [40].

In this paper we will show results obtained by SEM, TEM, and electrical resistance monitoring during post-growth annealing of Cu–C FEBID material from Cu(II) and Cu(I) precursors with respect to nanostructural changes and conductivity showing the potential of fabricating pure copper nanodots, from the as-grown amorphous Cu–C deposit. The thermal energy input favors the migration of Cu atoms to coalesce to pure Cu nanocrystals being dispersed inside and on top of the carbonaceous matrix.

## Experimental

### FEBID

The experiments were performed using a Hitachi 3600 scanning electron microscope (SEM) with a tungsten filament. The precursors were filled into their reservoirs inside a glove box in argon or dry nitrogen atmosphere. The deposition process has been carried out at room temperature on two types of substrates: Si with a 200 nm top layer of SiO_2_ and copper TEM grids with holey carbon films. The beam energy was fixed to 25 keV.

In this study bis(hexafluoroacetylacetonato)copper(II) [Cu(hfac)_2_, Cu(HC_5_O_2_F_6_)_2_] was used as a precursor and compared to earlier experiments with the precursors vinyltrimethylsilane copper(I) hexafluoroacetylacetonate, [(hfac)CuVTMS, (C_6_H_12_Si)Cu(HC_5_O_2_F_6_)], and dimethylbutene copper(I) hexafluoroacetylacetonate [(hfac)CuDMB, (C_5_H_12_)Cu(HC_5_O_2_F_6_)] [41].

The precursor flux was estimated to be about 10 monolayers per second for Cu(hfac)_2_. The exposure parameters for Cu(hfac)_2_ for 1 µm × 1 µm square deposits were: dwell time of 1 µs, pixel distance of 0.4 nm, and frame repetitions varying from 100 to 1000 with refreshment times of 0.625 s. The beam current was 0.4 nA. This corresponds to doses of 0.25 nC/µm^2^ (100 repetitions) and 2.5 nC/µm^2^ (1000 repetitions). For the 15 μm long lines we used 100 µs dwell time per pixel, 0.5 nm pixel distance, and 300 line repetitions with a refreshment time of 3 s. The beam current was 1 nA, which corresponds to the dose of 9 nC/µm^2^ and exposure time of 900 s. Tip deposits were obtained by the stationary dot exposure mode exposing a pixel for two minutes. Line and freestanding-rod deposits with (hfac)CuDMB and (hfac)CuVTMS from earlier FEBID experiments [39–40] were performed with a single scan at around 30 nm/s with 600 pA.

### Annealing

**Figure 1:**
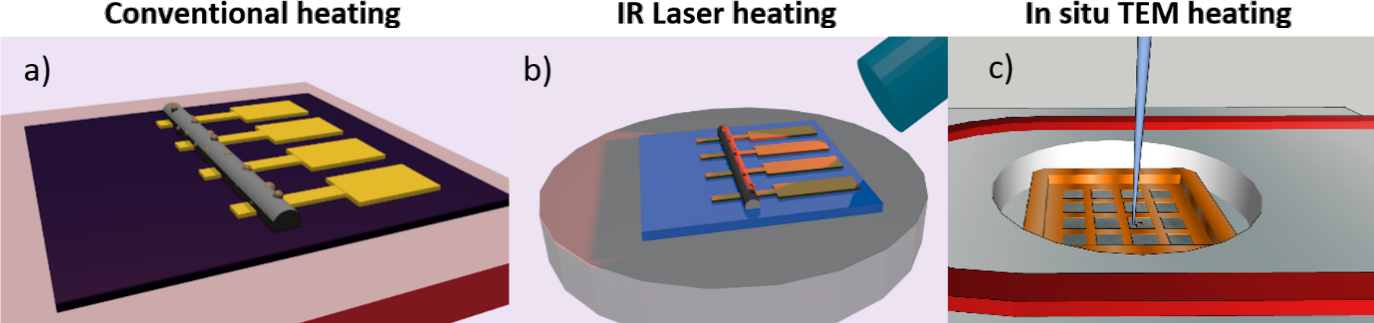
Sketch of post-growth annealing experiments: a) conventional heating using a hot plate in an SEM, b) SEM integrated laser heating; the substrate is heated by the IR laser pulses, c) post-growth thermal annealing during in situ TEM analysis. Conventional and IR laser annealing experiments were combined with in situ four-point probe resistance measurements.

Post-growth annealing in vacuum was achieved by various setups shown in Figure 1. The conventional heating using a hot plate was performed without breaking the vacuum after the deposition process. The experiments were performed inside the SEM for a temperature range from room temperature up to 220 °C. The sample was supported in a custom-made massive copper block on a resistive heater (Boraelectric). The control of temperature was done with a thermocouple directly coupled to the base of the substrate. During the heating we observed an increase of the pressure inside the SEM chamber from 5 × 10^−6^ to 5 × 10^−5^ mbar. We cannot exclude possible reactions of the deposits with the residual gas (most likely water and residual hydrocarbons) but we judge the influence as negligible as TEM annealing experiments at pressures better than 1 × 10^−6^ mbar also resulted in nanocrystal formation (see below in Figure 6).

An infrared laser system was integrated into the SEM chamber for in situ post-growth annealing directly after the deposition. The system was composed of a SvetWheel’s MU40-960-01 laser diode module and a fast diode current modulator VFM 20-25s (Messtec Power Converter GmbH) triggered by a signal function generator. The wavelength of emitted photons was equal to 960 nm (corresponding to an energy of 1.29 eV) with a total maximum laser power of 29 W. The tip of the laser optical fiber was placed in close vicinity of the FEBID deposit. The laser power, pulse duration and its frequency were adjustable by the signal generator. The size of the laser spot on the substrate was estimated to 65 µm × 130 µm, based on the molten area in SiO_2_ using the maximum laser power and a high pulse frequency.

The substrate surface temperatures generated by the laser during post-deposition annealing were determined experimentally and theoretically. Numerical finite element simulations with the annealing conditions (peak laser power of 13.6 W, pulse duration of 10 μs and frequency of 10 kHz) gave a stationary substrate surface temperature at the center of the laser beam of 158 °C which was reached after approximately 30 s. This was in good agreement with the value of 150 °C deduced from in situ four-point-probe resistance measurements of a gold wire deposited by e-beam lithography and PVD metal lift-off together with the pre-structure of the four-point electrodes.

TEM in situ annealing of FEBID deposits from Cu(hfac)_2_ was performed with a Gatan double tilt heating holder (Model 652) in a JEOL-2200FS microscope. The samples were heated up to 220 °C with a ramp rate of 20 K/min.

### Characterization

For SEM observation we used a Hitachi S4800 electron microscope and for the EDX measurements a Brooker/Oxford device mounted on a TESCAN LYRA microscope. EDX measurements were performed at 5 kV and 2 nA over 30 s with a 300 nm × 300 nm scan area on the squares to account for in homogeneities in the lateral copper nanocrystal precipitation. Standard EDX software was used to calculate the composition from the spectra.

High-resolution TEM (HR-TEM) images were taken at 200 keV. Selected area diffraction (SAD) was taken using the second smallest selected area aperture corresponding to an area of 400 nm in diameter on the sample. Chemical mapping was obtained using electron energy loss spectroscopy (EELS) operated in the scanning TEM (STEM) mode. The Cu K edge (928 eV) and a signal energy window of 40 eV (920–960 eV) after background subtraction were used to map the composition distribution of Cu, see Figure S1 in Supporting Information File 1.

The changes of resistance during the annealing were monitored by four-point probe measurements using an SEM-integrated 15-stage nanomanipulator from SmarAct. Conductive microprobes were connected via an SEM feedthrough to a Keithley 2400 Sourcemeter, with a source current of 100 µA and a voltage compliance level of 500 mV.

## Results and Discussion

### As-deposited material

Energy dispersive X-ray (EDX) analysis showed that at room temperature deposited Cu–C lines and squares obtained from Cu(II)(hfac)_2_ had an atomic ratio of approximately Cu/C/O/F = 10:64:25:1 with standard deviations of ±1 atom % for Cu, ±2 atom % for C, ±1 atom % for O, and ±0.3 atom % for F on eight deposits. This amounts to 10 ± 2 atom % of Cu. With respect to the stoichiometric copper content in the Cu(hfac)_2_ precursor of 3.7 atom % (disregarding the hydrogen) this was 2.7 times more copper in the deposit. The Cu content in deposits obtained from the (hfac)Cu(I)VTMS and (hfac)Cu(I)DMB precursors was about twice as high [41].

**Figure 2:**
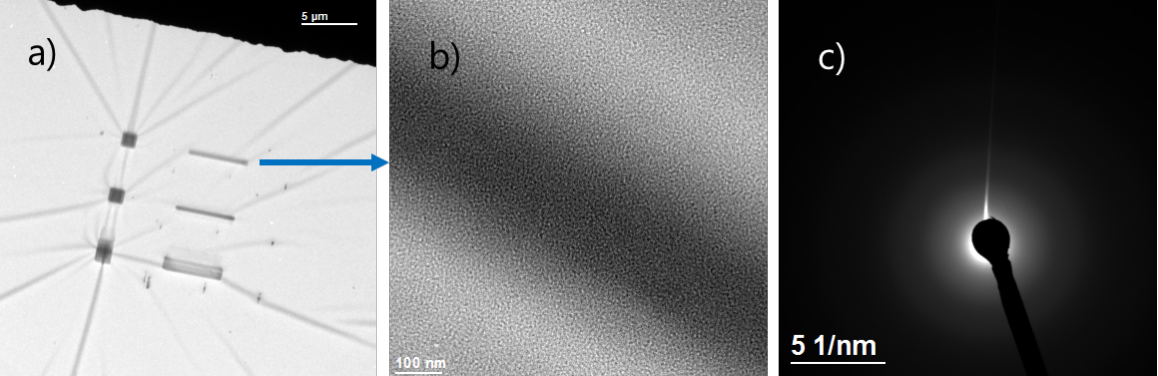
TEM of as-deposited lines and squares from Cu(hfac)_2_ on an amorphous carbon membrane on a TEM grid. a) An overview of deposits, b) zoom into a line deposit, c) a SAD pattern showing amorphous nature of the deposit.

Transmission electron microscopy (TEM) showed that dot, square, and line deposits from Cu(II)(hfac)_2_ on an amorphous carbon membrane were amorphous (Figure 2).

In contrast, as-deposited freestanding rods from earlier FEBID experiments with (hfac)Cu(I)VTMS showed small Cu nanocrystals homogeneously dispersed in a polymeric carbonaceous matrix (see below in Figure 7 taken with a Philips EM-430 TEM at 300 keV). This matrix contains all the ligand elements: carbon, oxygen, fluorine, and silicon as well as probably some hydrogen (not detectable by EDX) [42]. The difference between amorphous and nano-composite materials obtained for the Cu(II)(hfac)_2_ and (hfac)Cu(I)VTMS precursor, respectively, can be attributed to the lower thermal stability of (hfac)CuVTMS which is 63 °C compared to 250 °C for Cu(hfac)_2_.

Electrical measurements showed that the as-deposited FEBID lines from Cu(hfac)_2_ were highly resistive with a value of a few gigaohms being around the measurement limit of the Keithley device. This is in line with our former experiments which showed for all three copper precursors nonconductive behavior for the room temperature as-deposited material [41].

### Annealed material

#### SEM observations

**Figure 3:**
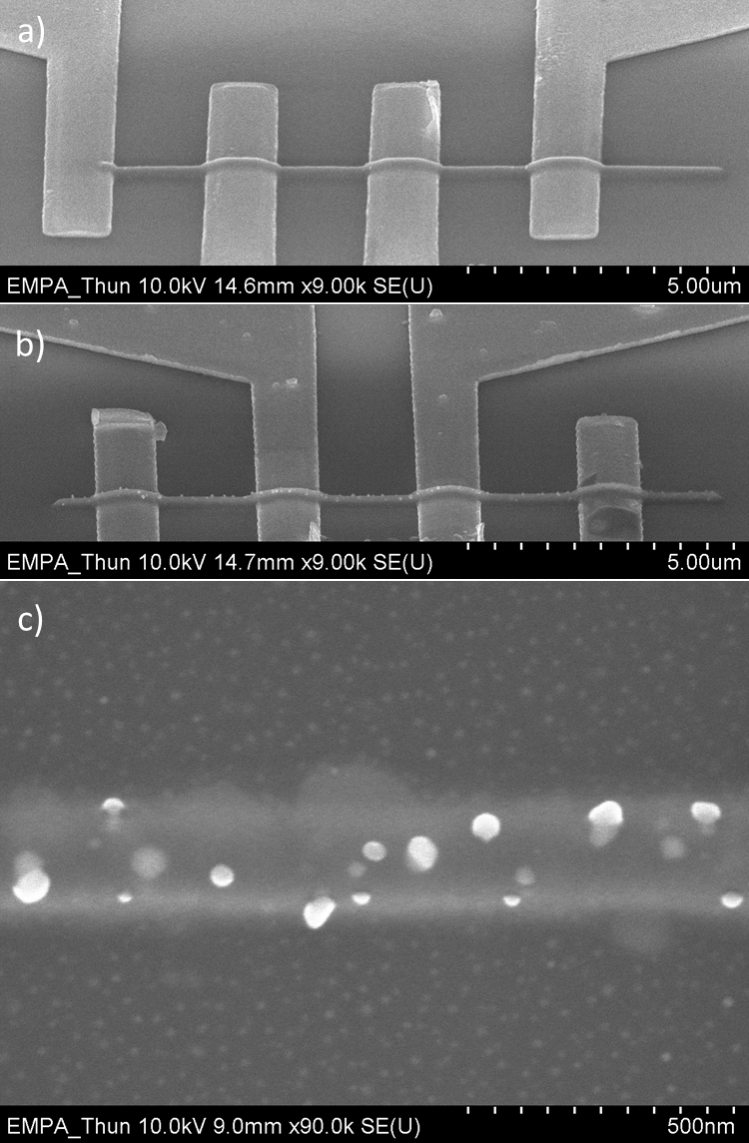
Post-growth annealing of FEBID line from Cu(hfac)_2_ between four gold electrodes. SEM tilt images (60°) of a) as-deposited line, b) after annealing at 200 °C for 30 min, and c) top-view zoom into central part of the annealed line showing the Cu nanocrystals on the line deposit and inside as well as on the halo deposit.

**Figure 4:**
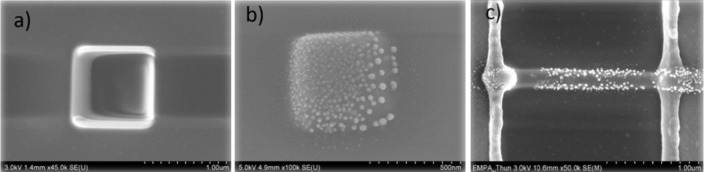
Post-growth laser annealing of FEBID deposits from Cu(hfac)_2_. SEM top view images of a) as-deposited square, b) Cu nanocrystals forming due to laser annealing at a power of 13.6 W over 1 min, c) laser-annealed FEBID line between two gold electrodes.

Upon annealing conventionally or with a laser the flat morphology of square, line, or tip deposits on the pre-patterned SiO_2_/Si substrate changes (Figure 3 and Figure 4). While the laser allows for local heating, the conventional hotplate approach allows for more accurate temperature measurements. The visible onset of Cu nanocrystal precipitation on the deposit surface starts at around 150 °C for the Cu(hfac)_2_ deposits on the pre-patterned SiO_2_/Si substrate. Further heating to about 200 °C for 30 min did not visibly change the appearance of the Cu nanocrystal precipitation.

EDX analysis after conventional heating to 200 °C for 30 min showed an atomic ratio of approximately Cu/C/O/F = 12:75:13:0 with standard deviations of ±2 atom % for Cu, ±2 atom % for C, and ±1 atom % for O on the same deposits. The 12 ± 2 atom % Cu content in the deposit is thus rather constant compared to the as-grown sample value of 10 ± 2 atom % Cu within the error limits and may have even been slightly overestimated systematically by the standard EDX software due to the surface-precipitated Cu nanocrystals corrected for absorption. Compared to the as-deposited material the fluorine completely disappeared and the oxygen content was halved. An estimation of the amount of precipitated copper nanocrystals that are visible in SEM gave only about 9 to 17 wt % (see Supporting Information File 1). Compared to the average Cu content in the as-grown and deposited samples of roughly 11 atom % (corresponding to an average 37 wt % Cu) this means that 20 to 28 wt % or 5 to 7 atom % of the copper dispersed in the matrix did not precipitate to be visible in SEM or segregated during the annealing. The matrix deposit volume shrunk during annealing to about 70% of its initial volume pointing to reticulation of the carbon network after release of fluorine and oxygen.

Interestingly, the size of the nanocrystals is much smaller in the thin halo region of the deposited line, yet the nanocrystal density is much larger. This may be due to the small amount of deposit material available to form the Cu nanocrystals but could be also due to the low irradiation dose of these regions and hence a slightly less reticulated carbon network which facilitates segregation at many places.

For laser-induced heating we found the same phenomenon of surface precipitation of Cu nanocrystals although the annealing time was only one minute. Also the same trend in the variation of the atomic ratio with annealing was found. Energy dispersive X-ray measurements of the deposit shown in Figure 4a and Figure 4bgave an atomic ratio of Cu/C/O/F = 9:52:32:6 for the as-grown FEBID material which changed to Cu/C/O/F = 10:66:23:0.6 after laser annealing. Considering the error limits of 2 atom % there is no fundamental difference to the trends and absolute values for Cu stated for conventional annealing. The Cu/C = 1:6.6 ratio after laser annealing of 1 min at around 158 °C (see section Experimental) is smaller than for thermal annealing, however, time and annealing temperature were lower for the laser than for thermal annealing. Figure 4 c shows that precipitation of Cu nanocrystals is not fully uniform across the line length in contrast to the conventionally annealed lines in Figure 3b and Figure 3c (see also Figure S5 in Supporting Information File 1). At the vicinity to the gold electrodes there are fewer nanocrystals visible which might be due to a varying distribution of the laser-induced temperature.

**Figure 5:**
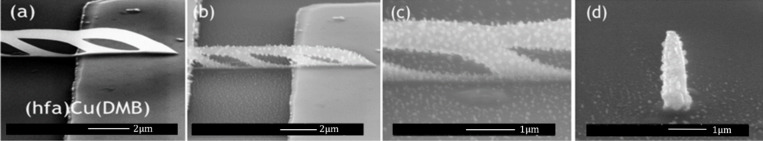
Periodic 3D FEBID line deposits from (hfac)Cu(DMB) between gold electrodes on SiO_2_/Si. SEM tilt images: a) before annealing, b) after in situ vacuum annealing, c) zoom on Cu nanocrystals, d) tip deposit showing also Cu nanocrystal formation.

The precipitation of Cu nanocrystals on the initial deposit surface was observed also in former experiments with the Cu(I) precursors. Figure 5 shows SEM tilt views of a periodic three dimensional line deposit obtained from (hfac)Cu(DMB). Such periodicity can arise when the vertical deposition rate is comparable to the scan speed of the focused electron beam. For the annealing experiments this is not of importance (for more details we refer to Bret et al. [43]). Upon annealing the same precipitation at deposit surfaces and at halo regions due to forward and backscattered electrons can be seen (Figure 5a–c). For a tip deposit the same features develop upon heating pointing to the fact that the dwell time per pixel during FEBID is not a very sensitive parameter for Cu nanocrystal precipitation (Figure 5d). The prolonged irradiation of a few minutes in the spot mode during FEBID with 20 keV electrons does not seem to change the reticulation of the matrix in such a way that formation of Cu nanocrystals remains contained inside the matrix. On the other hand, post-irradiation experiments with electrons energy of 5 keV were shown to be already effective to change the electron transport mechanisms in Pt–C and W–C FEBID deposits [27–29].

#### TEM observations

**Figure 6:**
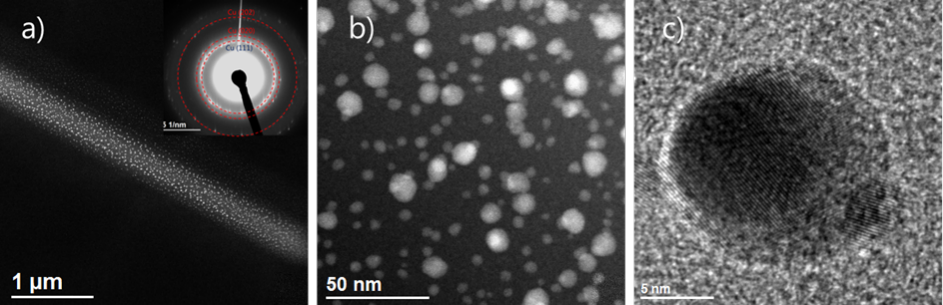
In situ TEM annealing for 10 min at 220 °C on a line deposit from Cu(hfac)_2_ shown in Figure 2. a) STEM high angle annular dark field (STEM-HAADF) image of Cu nanocrystals forming from the deposit material. b) HAADF image showing distribution of Cu nanocrystals. c) High-resolution TEM (HR-TEM) image of 15 nm sized polycrystalline Cu precipitate. The inset shows the SAD pattern of Cu fcc nanocrystals.

In Figure 6, the results of in situ TEM annealing experiments performed on the line and square deposit material from Cu(hfac)_2_ FEBID (shown in Figure 2) are presented. During annealing the deposits were not observed except for one to capture the temperature of crystal formation in a video.

The formation of nanocrystals took place at a temperature of 200 °C. The time for Cu nanocrystal formation was below 500 ms – the time range of the CCD camera to take a video frame image. After this rapid transition the nanocrystal arrangement did not change anymore. We compared it to regions that were not observed in TEM and found no differences so that the given temperature for crystal formation was not subject to irradiation during the video. We have tried different annealing rates and taken TEM images at different beam currents (which can be easily done by spreading the electron beam) and the results remained the same. In Figure 6 it can be seen that the amorphous material from Cu(hfac)_2_ turns to nano-composite with Cu nanocrystals embedded in an amorphous carbonaceous matrix. The size ranges from 2 nm to 20 nm for the face centered cubic (fcc) copper nanocrystals. This underlines that post-growth annealing has the potential to achieve one-digit-nanometer sized copper nanocrystals when using a high-resolution electron microscope for smallest dot deposition [44].

Theoretically, annealing a hemispherical FEBID deposit having a radius *r*_met_ and containing a given weight percentage *w*_met_ to pure metal would result in a metal hemisphere of radius

[1]
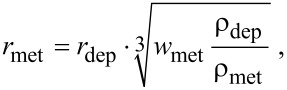


with ρ_met_ and ρ_dep_ being the densities of the metal and deposit, respectively. For our deposits from Cu(hfac)_2_ Equation 1 yields *r*_met_ = 0.46·*r*_dep_ with *w*_met_ = 0.35 (corresponding to the 10 atom % Cu in the as-grown deposit, see section “As-deposited material”), ρ_dep_ = 2.5 g/cm^3^ (from [45]) and ρ_Cu_ = 8.9 g/cm^3^. This means that 1 nm Cu dots could be annealed out of a 2 nm sized hemispherical FEBID material from Cu(hfac)_2_.

**Figure 7:**
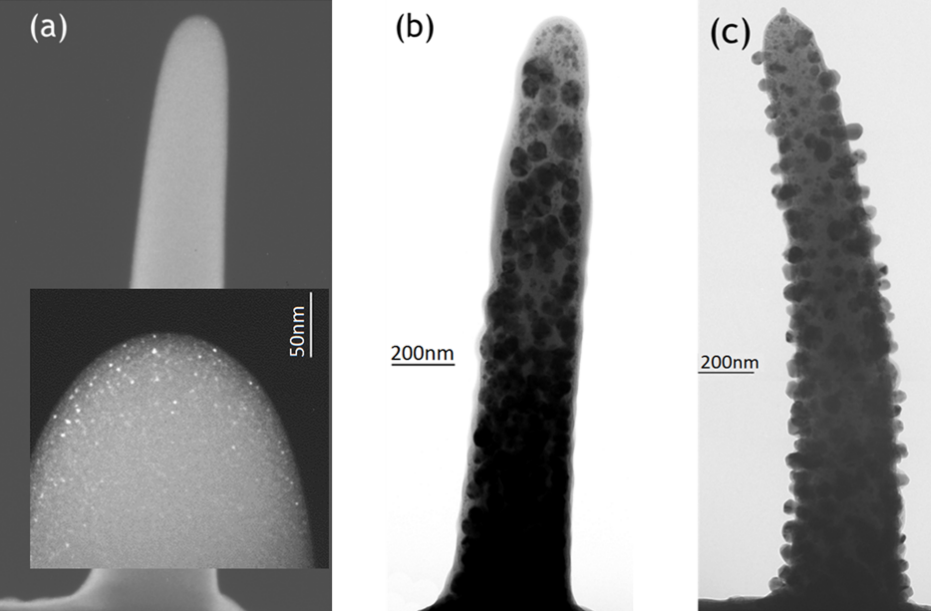
TEM in situ annealing of FEBID rods grown from (hfac)Cu(VTMS). a) Dark field image of an as-deposited freestanding rod. Inset: rod apex with small Cu nanocrystals in carbonaceous matrix. b) Bright-field image of same rod after 270 °C annealing and continuous TEM observation (200 keV). Large Cu nanocrystals form inside the rod. c) Bright field image of another rod not observed during the same annealing process. Cu nanocrystals form at the outside surface of the rod.

Selected results of earlier in situ TEM annealing experiments within a Philips EM-430 TEM on freestanding FEBID rods obtained from (hfac)Cu(VTMS) are shown in Fsigure 7. Interestingly, the comparison of Figure 7b and Figure 7c shows that the place of Cu nanocrystal precipitation can be controlled by post-irradiation with the high-energy electrons of a TEM in contrast to the experiments with Cu(hfac)_2_. The Cu crystals segregate around 140 °C inside the carbonaceous matrix when the deposit was exposed to the 300 keV electrons of the TEM during the annealing experiment, probably due to an electron-triggered reticulation of the polymeric carbonaceous matrix. The stronger reticulation of the carbon matrix seems to suppress the long-range mobility of Cu atoms to diffuse to the outside surface. In contrast, irradiation with only 20 keV is not efficient in reticulating the matrix in such a way that out-diffusion is hampered as was observed for (hfac)CuDMB deposits in Figure 5 and for Cu(hfac)_2_ deposits in Figure 3 and Figure 4.

In contrast, purification by electron beam induced heating of freestanding rods obtained from FEBID with (hfac)CuVTMS [42] gave much larger pure copper crystals (up to 150 nm in size). In comparison to the above results the differences may be explained by invoking an electron stimulated desorption effect which was suggested for FEBID by van Dorp et al. [46] which would facilitate the desorption of carbonaceous fragments and thus the formation of larger copper nanocrystals.

#### Electrical measurements

**Figure 8:**
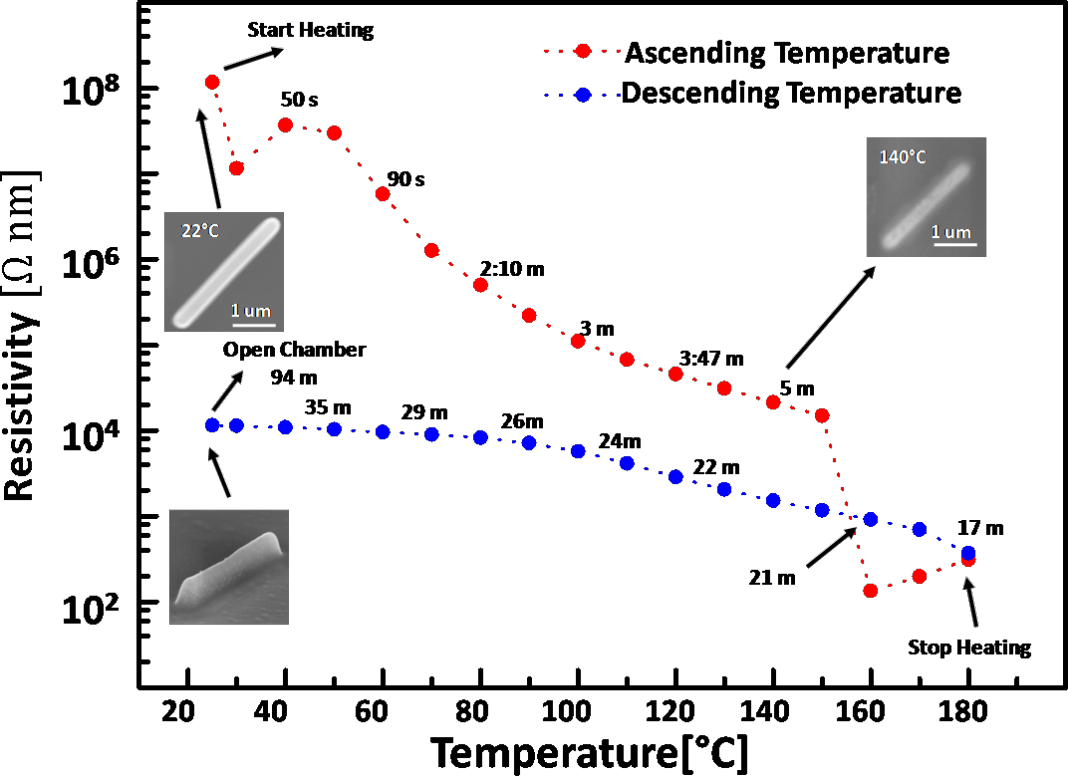
Calculated resistivity from the resistance measurement of a Cu–C line during in situ post-growth heating with a hot plate (red dots) and cooling down (blue dots) inside the SEM chamber. The resistance did not change when opening the chamber. The top SEM images show the morphology changes of an adjacent FEBID line which was observed simultaneously during the in situ resistance measurement.

Figure 8 shows the typical behavior of a Cu–C FEBID line for a heating/cooling cycle. The as-deposited lines were non-conductive, showing an electrical resistance of few gigaohms at room temperature. After thermal annealing the resistance dropped by four to five orders of magnitude to hundreds of kiloohms. At the current densities, used in the experiments (less than 0.1 MA/cm^2^) the migration process of Cu atoms is rather slow. Gazzadi and Frabboni [47] reported grain formation and electromigration in Pt–C material at current densities approaching 10 MA/cm^2^ which is about a factor 100 below our maximum current densities applied. A large resistance drop was observed after nanocrystals precipitated on the surface around 150 °C.

An increase in resistivity of one order of magnitude can be observed upon the cooling cycle from 180 °C to 25 °C. The temperature coefficient for bulk Cu is 0.00386 K^−1^ and would amount to an increase of the resistance by a factor of 1.6 only for the temperature difference of 155 K. The thermally activated transport observed in this case, can be due to the variable range hopping mechanism [48], corresponding to the insulating transport regime for granular materials, as it was observed previously for various FEBID deposits, composed of metallic grains embedded in carbonaceous matrix [49]. More detailed studies are planned to characterize the electronic transport of this material including the question whether the resistance-vs-temperature behavior of the samples would be stable. Here we focused on a proof of concept study.

## Conclusion

We have shown that nanostructural changes were induced in Cu–C FEBID material from Cu(I) and Cu(II) precursors upon post-growth annealing causing the segregation and precipitation of Cu nanocrystals. No fundamental differences between laser induced heating and conventional heating in the SEM and TEM with respect to trends and composition values were observed. As deposited the Cu–C FEBID deposits obtained from Cu(hfac)_2_ were amorphous while nanocomposite deposits were obtained from (hfac)CuVTMS and (hfac)CuDMB. The as-deposited materials were non-conductive. The transition into conductive material as well as the segregation and precipitation of the copper atoms occur upon conventional or laser heating. The surface precipitation of copper nanocrystals upon annealing opens a route for depositing pure Cu nanodot patterns using highly focused electron beams.

## Supporting Information

Supporting Information features additional information about the chemical mapping with electron energy loss spectroscopy, the estimation of Cu precipitation on deposit, and the distribution of Cu nanocrystals along the Cu–C lines after conventional and IR laser thermal annealing.

File 1 Additional experimental data.
