# Correction: Formation of pure Cu nanocrystals upon post-growth annealing of Cu–C material obtained from focused electron beam induced deposition: comparison of different methods

**DOI:** 10.3762/bjnano.6.196

**Published:** 2015-09-21

**Authors:** Aleksandra Szkudlarek, Alfredo Rodrigues Vaz, Yucheng Zhang, Andrzej Rudkowski, Czesław Kapusta, Rolf Erni, Stanislav Moshkalev, Ivo Utke

**Affiliations:** 1 Empa, Swiss Federal Laboratories for Materials Science and Technology, Laboratory for Mechanics of Materials and Nanostructures, Feuerwerkerstrasse 39, 3602 Thun, Switzerland; 2 AGH University of Science and Technology, Academic Centre for Materials and Nanotechnology, al. A. Mickiewicza 30, 30-059 Krakow, Poland; 3 Center for Semiconductor Components, State University of Campinas, 13083-870, Campinas, SP, Brazil; 4 Empa, Swiss Federal Laboratories for Materials Science and Technology, Electron Microscopy Center, Überlandstrasse 129, 8600 Dübendorf, Switzerland; 5 AGH University of Science and Technology, Faculty of Physics and Applied Computer Science, Department of Solid State Physics, al. A. Mickiewicza 30, 30-059 Krakow, Poland

**Keywords:** Cu(hfac)_2_, Cu nanocrystals, focused electron beam induced deposition (FEBID), post-growth annealing of Cu–C material

In Figure 8 of the original article, the scale of the ordinate was wrong. The correct figure looks as follows:

**Figure 1:**
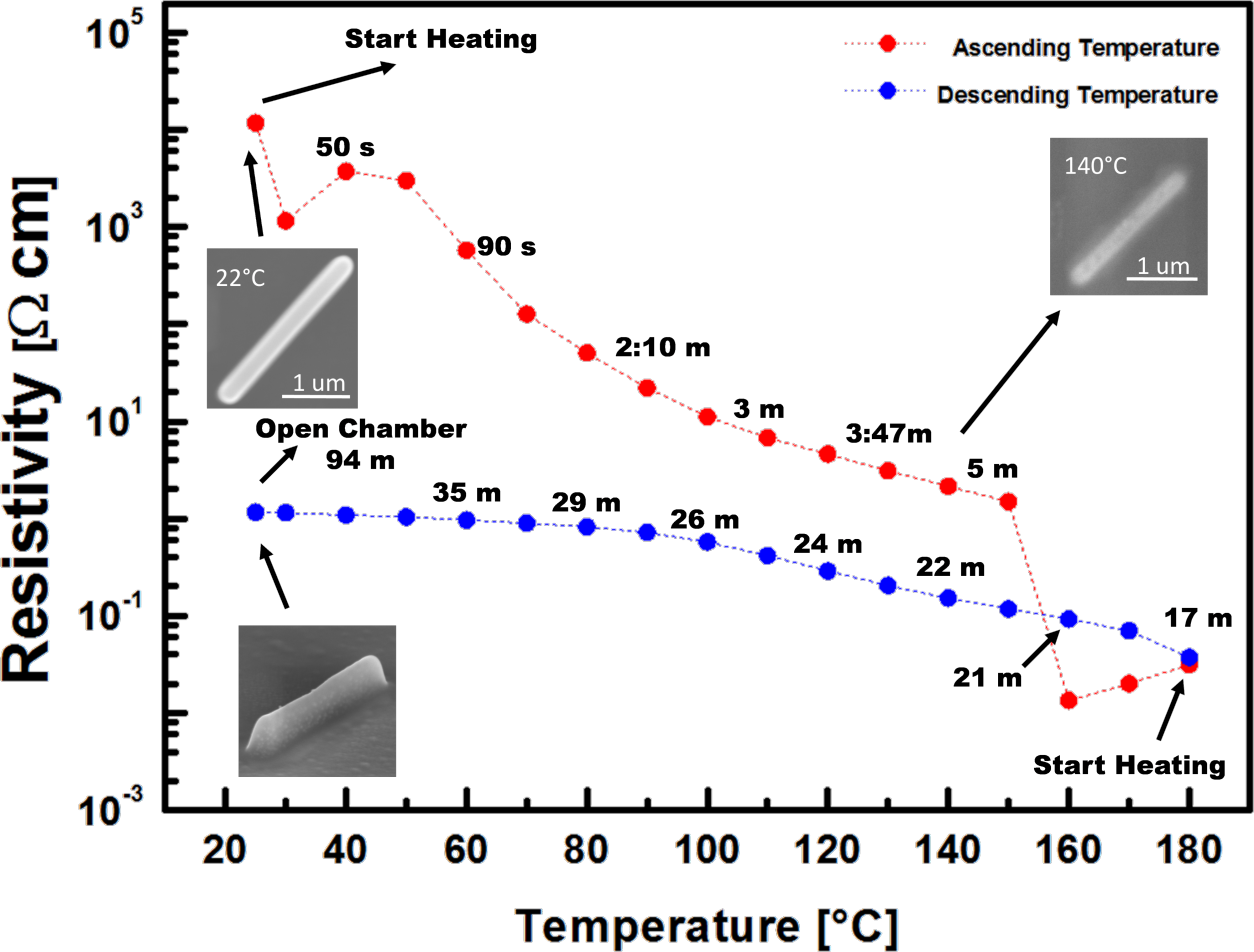
**Figure 8 in the original article:** Calculated resistivity from the resistance measurement of a Cu–C line during in situ post-growth heating with a hot plate (red dots) and cooling down (blue dots) inside the SEM chamber. The resistance did not change when opening the chamber. The top SEM images show the morphology changes of an adjacent FEBID line which was observed simultaneously during the in situ resistance measurement.

