# Supplementary material for: Formation of pure Cu nanocrystals upon post-growth annealing of Cu–C material obtained from focused electron beam induced deposition: comparison of different methods
Source: Beilstein J Nanotechnol. 2015 Jul 13;6:1508–17. doi: 10.3762/bjnano.6.156 (PMC4578412; doi:10.3762/bjnano.6.156)
Supplement: File 1 — Additional experimental data. [file Beilstein_J_Nanotechnol-06-1508-s001.pdf]

Supporting Information

for

Formation of pure Cu nanocrystals upon post-growth annealing of Cu–C material obtained from focused electron beam induced deposition: comparison of different methods

Aleksandra Szkudlarek[1,2], Alfredo Rodrigues Vaz[1,3], Yucheng Zhang[4], Andrzej Rudkowski[5], Czesław Kapusta[5], Rolf Erni[4], Stanislav Moshkalev[3] and Ivo Utke*[1]

Address: [1]Empa, Swiss Federal Laboratories for Materials Science and Technology, Laboratory for Mechanics of Materials and Nanostructures, Feuerwerkerstrasse 39, 3602 Thun, Switzerland; [2]AGH University of Science and Technology, Academic Centre for Materials and Nanotechnology, al. A. Mickiewicza 30, 30-059 Krakow, Poland; [3]Center for Semiconductor Components, State University of Campinas, 13083-870, Campinas, SP, Brazil; [4]Empa, Swiss Federal Laboratories for Materials Science and Technology, Electron Microscopy Center, Überlandstrasse 129, 8600 Dübendorf, Switzerland and [5]AGH University of Science and Technology, Faculty of Physics and Applied Computer Science, Department of Solid State Physics, al. A. Mickiewicza 30, 30-059 Krakow, Poland

Email: Ivo Utke* - ivo.utke@empa.ch

* Corresponding author

Additional experimental data

Chemical mapping with electron energy loss spectroscopy

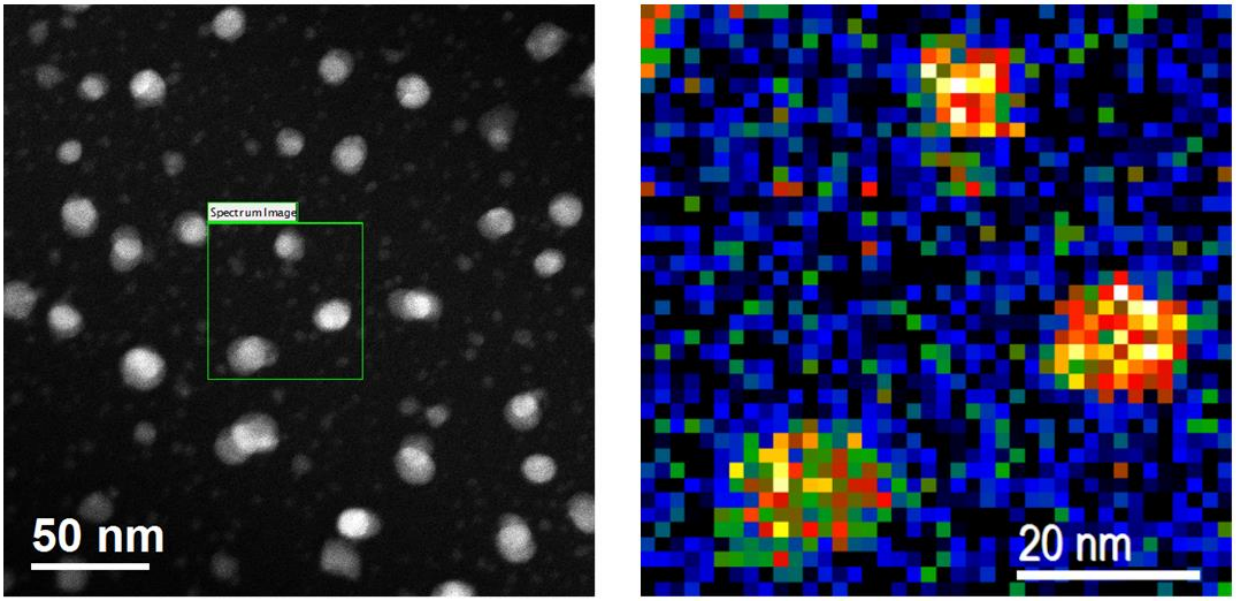

Figure S1: Chemical mapping with electron energy loss spectroscopy (EELS) operated in the scanning TEM (STEM) mode a) scan area, used for the analysis b) signal energy window of 40 eV (920–960 eV) after background subtraction were used to map the composition distribution of Cu visualized in false color. Blue, green, red and yellow represent an increasing Cu signal.

Estimation of Cu precipitation on deposit

During annealing copper nanoparticles precipitate on and close to the surface of the deposit. Before annealing the deposit dimensions were obtained by SEM imaging as shown Figure S2.

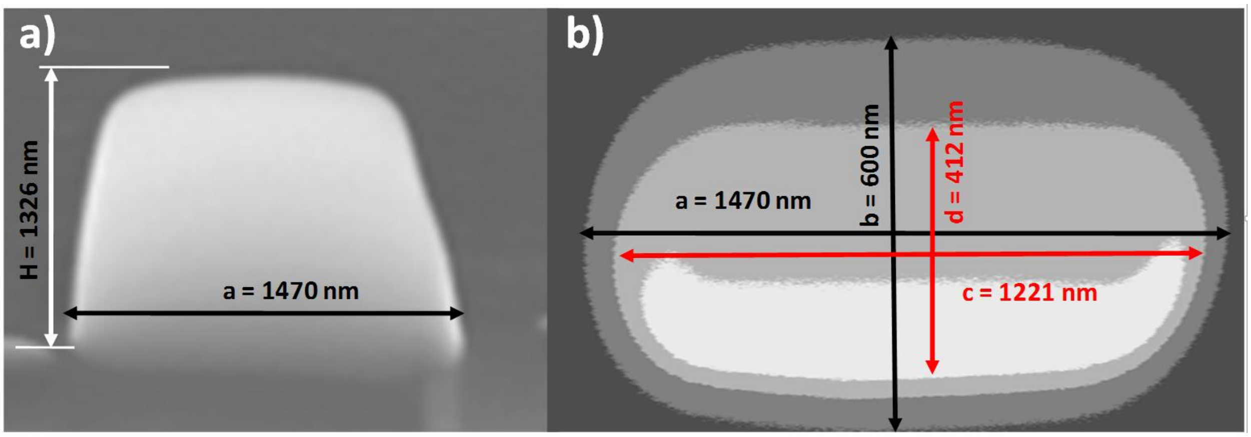

Figure S2: As-grown deposit with $Cu(hfac)_2$. FEBID exposure was performed with 1 μs dwell time, 0.4 nm pixel distance and 100 frame repetitions with a refreshment time of 0.625 s. The beam current was 0.4 nA. a) side view of deposit, b) top view contrast image of deposit.

The volume of deposit was approximated by a frustrated cone with elliptical base (Figure S3) the volume of which is given by Equation S1.

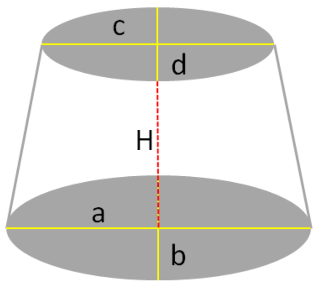

$$V = \frac{\pi}{12} \mathrm{H} \left((\mathrm{ab}) + (\mathrm{cd}) + \left(\sqrt{\mathrm{abcd}} \right) \right) \quad \text{(S1)}$$

Figure S3: Volume model of deposit.

The deposit was annealed at a temperature of 200 °C over 30 min in the SEM vacuum chamber. The visual analysis to count the number of Cu nanoparticles was performed from both sides of the deposit after annealing (Figure S4) and the quantity values of copper are shown in the Table S1.

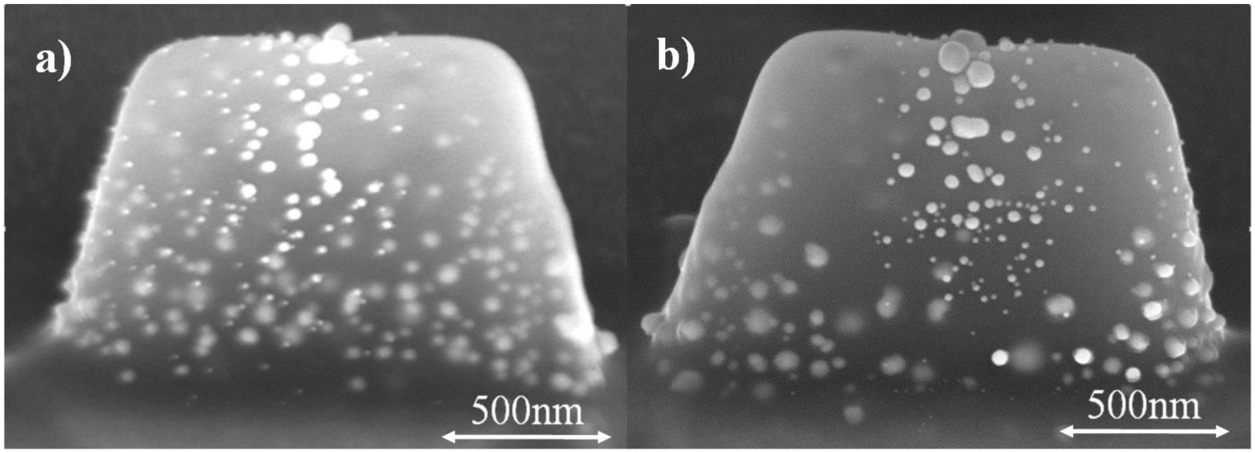

Figure S4: SEM tilt images of Cu precipitation on the surface and segregation close to the surface after annealing of the deposit shown in Figure S2. a) and b) show the front and backside of the deposit. The bright and clear contrast nanocrystals are on the surface while the slightly darker and blurred contrast nanocrystals are below the surface.

Table S1: Estimation of volume, mass, and weight percent (wt %) of visible copper nanocrystals. The matrix density value range was taken from Friedli, V.; Utke, I.; Mølhave, K.; Michler, J. *Nanotechnology* **2009**, 20, 385304. doi:10.1088/0957-4484/20/38/385304.

	Total	Copper	Carbonaceous Matrix
Volume [cm^3]	$7.12 \cdot 10^{-13}$	$1.6 \cdot 10^{-14}$	$7 \cdot 10^{-13}$
Density [g/cm^3]	—	8.56	1–2
Mass [g]	—	$1.3 \cdot 10^{-13}$	$7 \cdot 10^{-13}$ to $1.4 \cdot 10^{-12}$
wt % precipitated or segregated Cu	—	9–17	—

Distribution of Cu nanocrystals along the Cu-C lines after conventional and IR laser thermal annealing

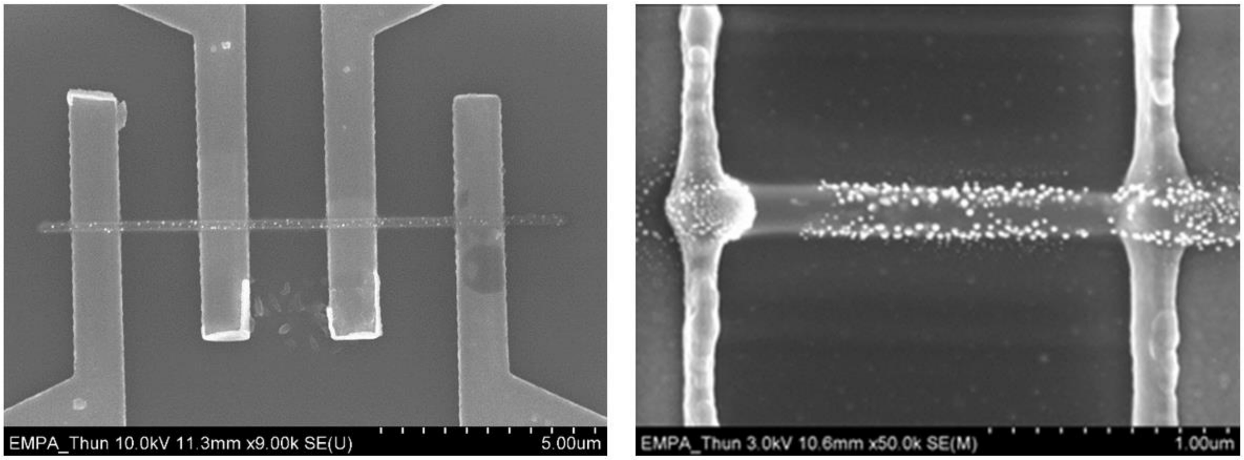

Figure S5: Comparison of distribution of Cu nanocrystals in Cu-C FEBID deposits after a) conventional annealing, and b) pulsed IR laser annealing.
